# 

**Published:** 1904-05-15

**Authors:** 


### ANNOUNCEMENTS.

THE Michigan State Dental Association will meet at Lansing, June 28th and 29th. There will be two whole days full of new and up-to-date dental tricks. No one that is within reach of this meeting can afford to miss it. Drop your work and come. Ride if you can, but walk if you must. The program will consist of eighteen practical papers and more than two dozen clinics, if we can get them; Le Gro says we can.

THE next annual meeting of the National Association of Dental Faculties will convene at 10 A. M., June 9th, 1904, in Washington, D. C., The executive committee will be in session the afternoon of June 8th, to consider such matters as may be brought before it. Arrangements are being made with the railroads for one and one-third fare on the certificate plan. The hotel as head-quarters, together with the railroad rates, will be announced later by circulars to the colleges.

H. B. TILESTON,
*Chairman Executive Committee.*

—✦—

THE Board of Dental Examiners of the State of Ohio will meet in Columbus, O., June 28th, 29th and 30th, at the Hotel Hartman, for examination of candidates for certificates of registration.

Application should be filed with the Secretary by June 18th.

For further particulars address,

H. C. BROWN, *Secretary*.
185 E. State Street.

—✦—

OFFICERS of the Chicago Dental Society for 1904-1905, elected at the annual meeting, April 5th, 1904: President, Thomas L. Gilmer; First Vice-President, C. N. Thompson; Second Vice-President, F. V. Yorker; Recording Secretary, Winthrop Girling; Corresponding Secretary, A. E. Morey; Treasurer, C. P. Pruyn; Librarian, J. H. Woolley; Member Board of Directors, J. G, Reid; Board of Censors, C. N. Johnson, Chairman, W. V-B. Ames, A. W. Harlan.

—✦—

THE forty-fifth annual meeting of the Northern Ohio Dental Association will be held in Cleveland, Tuesday, Wednesday and Thursday, June 7th, 8th and 9th. The program is a strong one and will be of exceptional interest to the general profession. The motto for the year is the ' Annihilation of Pain in Dentistry." Essayists and clinicians have been selected with this thought ever foremost. The best authorities and the most successful men in this line of work will be at this meeting. The members of the profession are cordially invited to attend. It is expected that we will have the largest attendance of any meeting ever held in this section of the country. You can not afford to miss it. COME.

W. G. EBERSOLE,

*Corresponding Secretary.*

—✦—

P. BLACKISTON'S SON & Co. sold over 15,000 copies of Gould's Medical Dictionaries last year and their total sales of these books amount to over 166,000 copies. What can have become of all of them?

—✦—

THE Board of Dental Examiners of Pennsylvania will conduct examinations simultaneously in Philadelphia and Pittsburg, June 8 to 11, 1904. Applicants for examination and license must address Hon. C. N. Schæffer, Secretary Dental Council, Harrisburg, Pa., for papers and further particulars.

—✦—

THE Committee on Railroad Accommodations for the East have made arrangements for fast through Pullman service to St. Louis from New York, with the Lackawanna R. R. Two special Pullman cars will leave New York Tuesday, August 23rd, at 10 A. M. The cost of our excursion, including berth each way, will be $35.50.

A proportionate reduction is made for those going from Buffalo, Toledo, Fort Wayne and cities on the line of, and connecting lines of the Wabash R. R. To those desiring to go in these special cars send notice to Charles A. Meeker, D. D. S., Secretary of the National Association, or to Guy Adams, General Passenger and Ticket Agent of the Delaware & Lackawanna R. R. Accommodations have been secured for the National Association of Dental Examiners at the Franklin Hotel, North-west corner of Sarah and Westminster Place, with rates from $1.50 to $6.00 per day, European plan.

Hotel first class. Secure accommodations by writing to E. C. Dunnavant, St. Louis Service Co., 7th and Olive streets, St. Louis, Mo.

CHARLES A. MEEKER, D. D. S., *Secretary.*

—✦—

THE Indiana State Dental Association meets at Indianapolis, June 14, 15 and 16, 1904.

—✦—

### FOURTH INTERNATIONAL CONGRESS.

The Committee on Local Information submit the following facts, important for the success of the Fourth International Dental Congress:

Every member of the profession who has at heart the welfare of dentistry should give it support, financially and by attendance.

All American members of the dental profession should keep in mind that this Congress will be held in our country, and pride should prompt us to make the meeting one of great magnitude and far excelling all predecessors. Let every dentist take hold with a will and push it to a successful issue.

This Congress should be of great interest to all members of the profession. There is planned a monster clinic from the best clinicians of the world; scientific papers from the pens of the most noted and scholarly men of the profession, and an exhibit to excell all others.

The meeting, clinics and exhibits will be held in the great Coliseum, where ample accommodations are secured.

Hotel Jefferson, the general headquarters of the Dental Congress, is one of the most fashionable and complete hotels in the West, if not in America, and is located on Twelfth Street, one block from the Coliseum. Hotels of Saint Louis will be sufficient to meet all requirements.

The Information Bureau of the Exposition has a list of ninetyseven well-established hotels now doing business in Saint Louis, with a capacity of 41,000 guests, at prices ranging from fifty cents a day up on the European plan, and from one dollar a day up on the American plan. These established hotels have been supplemented during the year 1903 by thirty-five new permanent hotels now opening, or about to open, increasing the permanent hotel capacity to 67,000 guests, at prices ranging from one dollar a day up. The exposition management holds the signed agreement of the leading hotels that "Rates shall not be increased during the World's Fair period." Prices are now lower in Saint Louis than in any other city for similar hotel accommodations and service.

The Exposition Information Bureau's list of 132 permanent hotels includes only the better class. There are now 173 hotels, large and small, in operation in the city, and the new hotel enterprises being inaugurated justify the belief that the number will reach 250 before the opening day of the World's Fair.

Besides hotels with accommodations for more than 200,000 guests, the Exposition Information Bureau has a list of boarding-houses and rooming-houses of respectable character, on the street-car lines, with lodgings for 65,000 guests, and a list of private houses that will let rooms for 20,000 persons.

All over the city, apartment-houses and rooming-houses are available for those who prefer rooms away from crowds, with meals at the restaurants.

There are 485 restaurants in Saint Louis that have a national reputation for good fare, good service, cleanliness and moderate prices. Twenty of these 485 restaurants can take care of 40,000 patrons.

The climate in Saint Louis is temperate in summer and most delightful in the Spring and Fall. It is the most central and accessible of the four large cities of the United States, twenty-seven railways entering it, and passenger steamers on the Mississippi River reaching it from North and South.

World's Fair cheap rates on railways and steamboats will be offered during the whole exposition season, as follows:

### RATES ADOPTED BY THE FOLLOWING ASSOCIATIONS:

### New England Passenger Association, Trunk Line Association, Central Passenger Association, and the South-Eastern Passenger Association.

Tickets will be on sale from April 15th.

Season tickets for eighty percent of double one fare, good to return until, December 15th.

For sixty days, one and one-third fares, not good to return after December 15th.

For ten days, one fare, plus $2.00, from points within two hundred and fifty miles of Saint Louis.

For fifteen days, one fare, plus $2.00, from points over two hundred and fifty miles from Saint Louis.

Saint Louis is the fourth city of the United States in point of population, having 750,000 people. Certainly no city is more attractive with interest for the student of nature, science, history, etc. There are twenty-four public parks, containing over 2,100 acres, all well improved.

The World's Fair grounds lie five miles from the river, on the western edge of the city, and are reached quickly and comfortably by steam railway and fast trolley lines.

The visitors reach the city through the largest and most beautiful railway station in the world. Thirty-two tracks run into the station side by side, and the midway, or glass-roofed hall in front of the gates to the trains, will hold 30,000 people. Most of the hotels, except those temporary ones near the World's Fair grounds, are within ten minutes' ride of the station, in the heart of the business district. Street cars, reaching all of the hotels for one fare, pass the station, and the cab, carriage and baggage system is excellent.

The great Music Hall and Coliseum building that is secured for the meetings of the International Dental Congress is down-town, on Olive street, between Thirteenth and Fourteenth streets, within easy walking distance of Union Station, the hotels and the business district. It has a seating capacity for 8,000 people, with section rooms that will be arranged for the various committees and exhibitors.

Naturally, every member attending the Fourth International Dental Congress will wish to see the World's Fair. This will be the most maguificent the world has ever seen, and it will probably be the last great exposition to be held in this country for many years, because of the enormous expenditure of labor and money attending it.

We appeal to all reputable, legally-qualified practitioners of dentistry to interest themselves in the success of this great international meeting.

**Let every dentist in America put forth his greatest efforts to advance the cause. Make application at once through your State Chairman for membership certificate. See that your fellow-practitioner is enrolled, and then, and not till then, will you have done your full duty.**

The membership fee to the Congress will be $10.00. It will entitle the holder to the official badge and all the rights and privileges of the Congress. He will also receive one copy of the transactions. Without a membership certificate it will be impossible to get into the general meetings, sections and clinics, or attend any of the various entertainments during the Congress.

This great Congress presents to the American dentist an opportunity to see and hear the brightest and most learned men of the profession from all parts of the world, men of international reputation that shine as clinicians, and men of great renown of the inventive turn of mind. This international meeting will be the Mecca of the profession of the world. We will all receive new light and be stimulated to a higher appreciation of our noble profession.

The local Committee of Arrangements and Reception, with its various minor committees, is working with increasing energy and will be ready to meet you and extend you a most hearty welcome.

Any further information can be obtained from the Committee.

D. O. M. LeCRON, Chairman, Missouri Trust Building,

MAX FENDLER, Secretary.

